# Obesity: a perfect storm for carcinogenesis

**DOI:** 10.1007/s10555-022-10046-2

**Published:** 2022-08-30

**Authors:** Benjamin H. L. Harris, Valentine M. Macaulay, David A. Harris, Paul Klenerman, Fredrik Karpe, Simon R. Lord, Adrian L. Harris, Francesca M. Buffa

**Affiliations:** 1 Department of Oncology, University of Oxford, Oxford, OX3 7DQ UK; 2 St Anne’s College, 56 Woodstock Rd, Oxford, OX2 6HS UK; 3 Nuffield Department of Surgical Sciences, University of Oxford, Oxford, OX3 9DU UK; 4 Peter Medawar Building for Pathogen Research, University of Oxford, Oxford, OX1 3SY UK; 5 Oxford Centre for Diabetes, Endocrinology and Metabolism, Department of Medicine, University of Oxford, Oxford, OX3 7LE UK; 6 Early Phase Clinical Trials Unit, Churchill Hospital, Oxford, OX3 7LE UK

**Keywords:** Obesity, Cancer, BMI, Adiposity, Carcinogenesis, Hallmarks of cancer

## Abstract

Obesity-related cancers account for 40% of the cancer cases observed in the USA and obesity is overtaking smoking as the most widespread modifiable risk factor for carcinogenesis. Here, we use the hallmarks of cancer framework to delineate how obesity might influence the carcinogenic hallmarks in somatic cells. We discuss the effects of obesity on (a) sustaining proliferative signaling; (b) evading growth suppressors; (c) resisting cell death; (d) enabling replicative immortality; (e) inducing angiogenesis; (f) activating invasion and metastasis; (g) reprogramming energy metabolism; and (h) avoiding immune destruction, together with its effects on genome instability and tumour-promoting inflammation. We present the current understanding and controversies in this evolving field, and highlight some areas in need of further cross-disciplinary focus. For instance, the relative importance of the many potentially causative obesity-related factors is unclear for each type of malignancy. Even within a single tumour type, it is currently unknown whether one obesity-related factor consistently plays a predominant role, or if this varies between patients or, even in a single patient with time. Clarifying how the hallmarks are affected by obesity may lead to novel prevention and treatment strategies for the increasingly obese population.

## Introduction

The pandemics of obesity and cancer are major worldwide healthcare challenges. By 2030, it is projected that ~ 50% of the adult population in the USA will be obese [1]. In parallel, the incidence of cancer is rising, with the lifetime incidence now estimated at approximately 1 in 2 (at least in the UK) [2] and obesity is set to be the main modifiable cause of cancer, overtaking smoking [3]. Obesity-related cancers already account for 40% of the USA cancer cases [4]. Obesity has been associated with an increased risk of cancer in a wide range of tissues [5] and absence of body fatness lowers risk of several cancers including breast (post-menopausal), bowel, endometrium, oesophagus, ovary, liver, gastric cardia, gallbladder, pancreas, kidney, meningioma, multiple myeloma and thyroid [6]. While this epidemiological data merely demonstrates an association, different cancers appear differentially affected, with causality in some cases backed by evidence from model systems. Supporting a causal relationship, we see that reduction of body mass following bariatric surgery is associated with a reduced cancer risk, particularly in obesity-related cancers [7]. And by 2035, it is estimated that ~ 40% of endometrial, > 25% oesophageal, > 20% renal and ~ 20% liver cancers globally will be attributable to having a high body mass index (≥ 25 kg/m^2^) [8]. Furthermore, obesity in general is associated with worse prognosis in patients with cancer, although the jury is still out in certain tumour types [9].

**Fig. 1:**
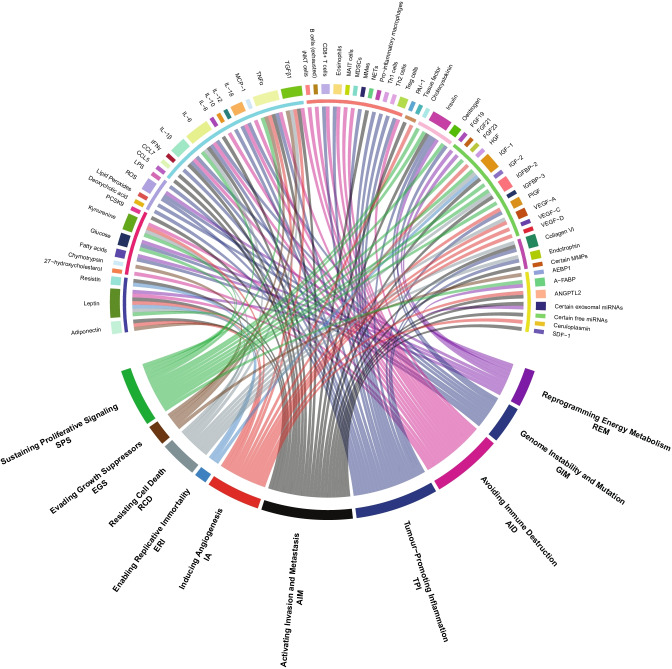
**How obesity can influence the hallmarks of cancer, enabling characteristics and emerging hallmarks.** A circos plot illustrating the links between obesity-related factors and the hallmarks of cancer (classical and emerging) and enabling characteristics. Obesity-related factors are located in the top half of the plot, while hallmarks of cancer and enabling characteristics are found in the bottom half. In the top half of the plot, the outer coloured blocks denote the factors that are affected by obesity with one colour per factor (e.g. cyan for adiponectin). The ten groupings in Table 1 are indicated by the inner semi-circle in the top half of the plot (e.g. adiponectin, leptin and resistin are all classified as adipokines in Table 1 and indicated by an indigo bar in the inner semi-circle). Lines between the bottom half and top half highlight obesity-related factors and their relationship to particular hallmarks/characteristics. The colour of the lines relates to the relevant hallmark/characteristic (e.g. green for sustaining proliferative signaling). Factors can influence several hallmarks. The basis for linking each factor to a particular hallmark/characteristic is discussed in the main text, while Table 1 gives the direction of change of each factor in obesity as well as an exemplar reference

There is no doubt that obesity changes body physiology, metabolism, proteome, transcriptome and epigenetic outlook in multiple tissues, and that these changes can be linked with many features of the carcinogenic process. In their seminal paper, Hanahan and Weinberg defined the ‘hallmarks of cancer’, viz., properties that mark cells out for the development and maintenance of tumour growth [10]. In this article, we discuss how these hallmarks and enabling characteristics might be affected by obesity, highlighting major and emerging commonalities, such as the link between inflammation in promoting tumour growth and the chronic inflammatory state observed in obesity [11]. Adipose tissue is also a major source of oestrogen, a potent anabolic hormone, and this is one of several growth factors raised in obesity. Notably, although this manuscript is structured around individual ‘hallmarks’, we find particular effectors (such as leptin, IL-6 and some hormones) affect multiple hallmarks/enabling characteristics and therefore appear in several sections (illustrated in Fig. 1 and Table 1). However, which obesity-induced factors predominate in tumorigenesis at different sites, or how local the effects of each factor are, is not well-defined. In this paper, we cover salient concepts and their basis in the literature. We touch on new therapeutic observations obtained when treating patients with obesity and the influence of obesity on prognosis. We highlight how the obesity-related factors potentially impact on multiple aspects of tumour development and contribute to a ‘perfect storm’ for carcinogenesis. However, such is the broad nature of the subject area, it is beyond its scope to discuss in depth every mechanism, experimental protocol and model system in all studies: these are available in the referenced material. Indeed, this field has its controversies, unanswered questions and new emerging hypotheses, and it is not possible to debate all of these within this piece but we do aim to signpost the reader towards key areas to stimulate further work.

**Table 1:** **Factors affected by the obese state. **Local and (potentially) systemic factors affected by the obese state, with the relevant hallmarks/enabling characteristics of cancer they may influence. Factors are grouped by type, and exemplar references are given. Downstream effectors related to these factors (e.g. TP53, ATR, AHR and HIF1α) are discussed in the main text. Key: *SPS*, sustaining proliferative signaling; *EGS*, evading growth suppressors; *RCD*, resisting cell death; *ERI*, enabling replicative immortality; *IA*, inducing angiogenesis; *AIM*, activating invasion and metastasis; *TPI*, tumour-promoting inflammation; *AID*, avoiding immune destruction; *GIM*, genome instability and mutation; *REM*, reprogramming energy metabolism

Factor	Direction in obesity	Associated hallmarks	Organism	Tissue	Exemplar reference(s)
Adipokines					
Adiponectin	↓	EGS, IA, AIM	Human	Plasma	[12]
Leptin	↑	SPS, RCD, ERI, IA, AID, REM	Human	Serum	[13]
Resistin	↑	ERI, AIM	Human	Serum	[14]
Dietary factors					
27-hydroxycholesterol	Postulated to be ↑	AID	Human	Serum	[15]
Chymotrypsin	Postulated to be ↑	EGS	Rat	Pancreas	[16]
Fatty acids (profiles need further elucidation)	↑	GIM, REM	Human	Serum	[17]
Glucose	↑	SPS, GIM, REM	Human	Plasma	[18]
Kynurenine	↑	SPS, RCD, IA, AID	Human	Plasma	[19]
PCSK9	↑	AIM	Human	Serum	[20]
Oncometabolites					
Deoxycholic acid	↑	TPI	Mouse models	Serum	[21]
Lipid peroxides (e.g. measured by malondialdehyde)	↑	GIM	Human	Serum	[22]
ROS	↑	GIM, TPI, REM	Rat	Colon/Kidney	[23]
LPS	↑	TPI	Human	Serum	[24]
Cytokines and cytokine-like proteins					
CCL5	↑	AIM	Mouse models	Lungs/Lymph nodes	[25]
CCL7	↑	AIM	Human	Periprostatic adipose tissue	[26]
IFNγ	↑	TPI	Human	Serum	[27]
IL-1β	↑	AIM, GIM, TPI, AID	Human	Adipose	[28]
IL-6	↑	SPS, RCD, AIM, GIM, TPI, AIM	Human	Plasma	[29]
IL-8	↑	AIM	Human	Serum	[30]
IL-10	↓	AID	Human	Serum	[31]
IL-12	↑	TPI	Human	Serum	[27]
IL-18	↑	GIM, TPI, AID	Human	Adipose/Plasma	[32]
MCP-1	↑	AID	Human	Serum	[33]
TNFα	↑	SPS, EGS, IA, AIM, TPI, AID	Human	Serum	[34]
TGFβ1	↑	EGS, RCD, IA, AIM, AID	Human	Plasma	[35]
Immune cells, markers and their products					
iNKT cells	↓	AID	Human	Omentum	[36]
B cells (exhausted)	↑	AID	Ex vivo	Adipose	[37]
CD8 + T cells	↑	TPI, AID	Mouse models	Adipose	[38]
Eosinophils	↓	TPI, AID	Mouse models	Adipose	[39]
MAIT cells	↓	AID	Mouse models	Adipose/Ileum	[40]
MDSCs	↑	AID	Human	Serum	[41]
MMes	↑	TPI	Human	Adipose	[42]
NETs	↑	AIM	Human	Serum	[43]
Pro-inflammatory macrophages	↑	TPI	Mouse models	Adipose	[44]
Th1 cells	↑	TPI	Mouse models	Adipose	[45]
Th2 cells	↓	TPI	Mouse models	Adipose	[46]
T_reg_ cells	↓	TPI, AID	Mouse models	Adipose	[47]
Proteins of the fibrinolytic system					
PAI-1	↑	AIM	Human	Adipose	[48]
Tissue factor	↑	IA	Mouse models	Various (incl. Adipose/Liver)	[49]
Hormones					
Cholecystokinin	↑	SPS	Mouse models	Pancreas	[50]
Insulin	↑	SPS, AIM, GIM, TPI, REM	Human	Plasma	[51]
Oestrogen	↑	SPS, GIM	Human	Plasma	[52]
Growth factors and their binding proteins					
FGF19	↓	Potentially REM	Human	Serum	[53]
FGF21	↑	REM	Human	Serum	[54]
FGF23	↑	SPS	Human	Serum	[55]
HGF	↑	IA	Human	Serum	[56]
IGF-1	Controversial	SPS, RCD, AIM			[57–60]
IGF-2	↑	SPS	Human	Serum	[61]
IGFBP-2	↓	SPS, RCD, AIM	Human	Serum	[62]
IGFBP-3	Controversial	SPS			[63, 64]
PlGF	↑	IA, TPI	Human	Serum	[65]
VEGF-A	↑	RCD, IA	Human	Serum	[66]
VEGF-C	↑	IA	Human	Serum	[66]
VEGF-D	↑	IA	Human	Serum	[66]
Extracellular matrix components and remodelling					
Collagen VI	↑	RCD, AIM, TPI	Mouse models	Adipose	[67]
Endotrophin	↑	RCD, TPI	Mouse models	Adipose	[67]
Certain MMPs e.g. MMP-2	↑	AIM	Cell lines		[68]
Other salient species					
AEBP1	↑	EGS	Mouse models	White adipose tissue	[69]
A-FABP	↑	SPS, REM	Human	Plasma	[70]
ANGPTL2	↑	IA, AIM	Human/Mouse models	Serum	[71]
Certain exosomal miRNAs e.g. miR-122	↑	AIM, REM	Mouse models		[72]
Certain free miRNAs e.g. miR-27a	↑	AIM	Cell lines		[73]
Ceruloplasmin	↑	IA	Human	Adipose/Plasma	[74]
SDF-1	↑	AIM	Mouse models	Adipose	[75]

## Sustaining proliferative signaling

**Fig. 2:**
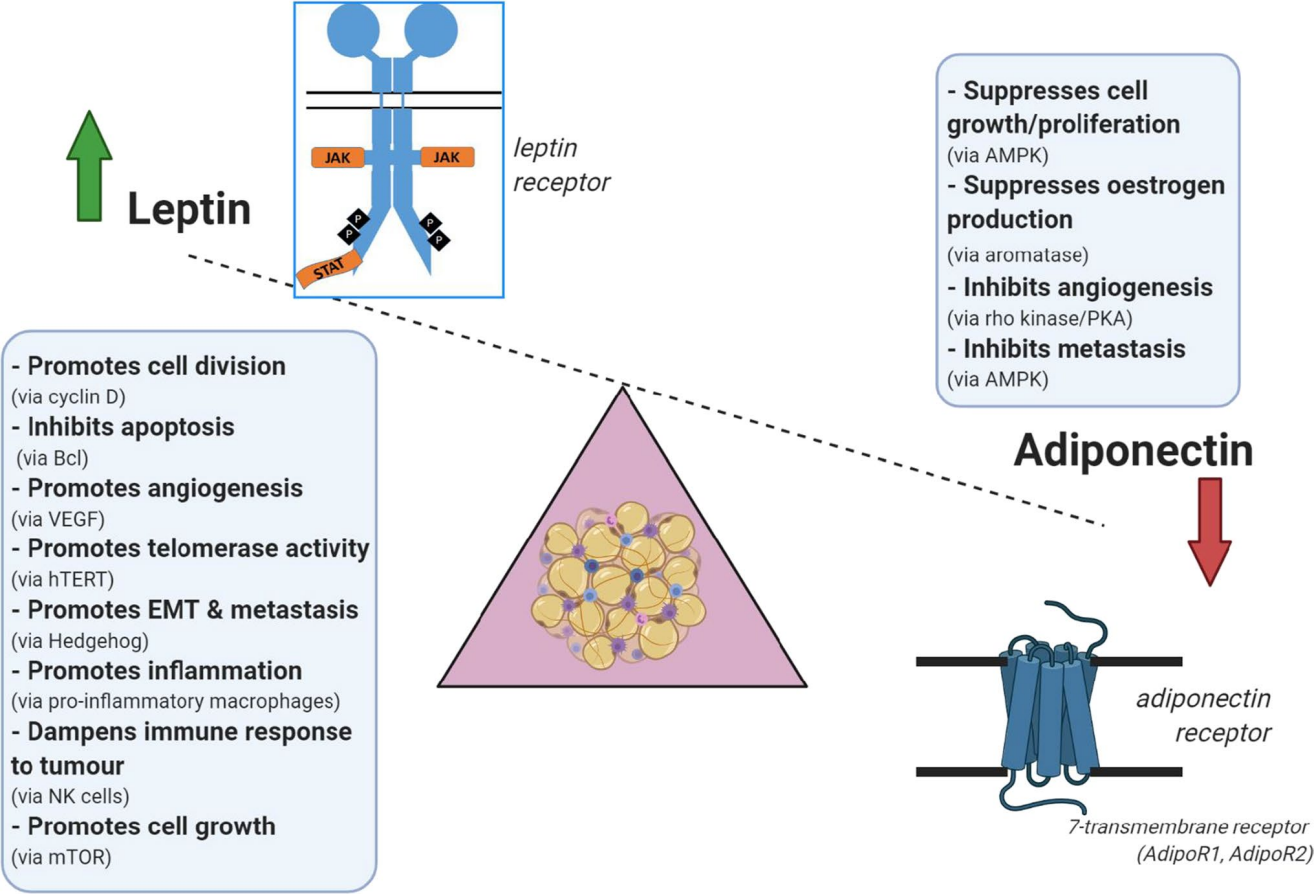
**Leptin/adiponectin balance in obesity. **Leptin and adiponectin are two adipokines produced by adipose tissue. Leptin levels rise in obesity, while adiponectin falls. Leptin binds to a growth factor receptor at the cell surface, and promotes cell proliferation/opposes cell death by a variety of mechanisms. These include promoting cell growth (via stimulation of mTOR), angiogenesis and metastasis, while at the same time inhibiting antitumour mechanisms such as apoptosis and immune surveillance. Overall, therefore, the actions of leptin are pro-tumorigenic. Adiponectin, in contrast, binds to a G-protein coupled receptor at the cell surface, and broadly opposes these actions of leptin, by activating kinases such as AMP kinase (which decreases mTOR activity) and protein kinase A (which decreases angiogenesis). Normally the actions of these two hormones are balanced, to maintain cell proliferation at an appropriate rate, but in obesity leptin concentrations rise, and adiponectin falls, promoting a tumorigenic environment

A fundamental property of cancer cells is their ability to divide and proliferate uncontrollably. In the obese state, blood leptin levels rise, as a result of the increased secretion from adipose tissue [13]. Circulating leptin helps to regulate appetite, insulin sensitivity and hormones such as thyroid stimulating hormone. However, leptin also has pro-oncogenic actions. Leptin is a growth factor, binding to a receptor tyrosine kinase (Ob-R/LepR), and activating the JAK/STAT, ERK and PI3K/Akt signalling pathways [76] (Fig. 2). In vitro, leptin promotes cell cycle progression by inducing the production of cyclin D1 which drives cells past the G1/S checkpoint in various cancer cell lines (ZR-75–1, Hep3B, HepG2) [77, 78]. However, leptin is not the only factor at play in obesity tumour growth, highlighted by recent work in pancreatic adenocarcinoma mouse models demonstrating accelerated tumorigenesis in obese mice deficient in leptin signalling with the possible involvement of cholecystokinin [50].

Another growth promoter, associated with increased leptin production, is oestrogen, whose role in breast cancer is well established. Notably, a major source of oestrogen is adipose tissue, where it is made by the enzyme aromatase, and serum oestrogens are raised in obese humans [52]. Oestrogen receptor status (ER) is a determinant of breast cancer treatment and influences prognosis. Oestrogen has also been linked to the development of endometrial and colorectal cancer in humans, although the colorectal relationship appears more complex [79, 80]. In post-menopausal women, hormone replacement therapy (oestrogen combined with progesterone) has been associated with reduced colorectal cancer risk [81]. However, recent work shows oestrogen (7-beta estradiol) alone increased mitosis but the combination of oestrogen and progesterone increases apoptosis in murine colon tumours [82]. Oestrogen also influences proliferation in pancreatic cancer cell lines (HTB147, CFPAC, Panc-1), related to the oestrogen receptor subtype ratio [83]. Furthermore, there have also been claims that the gut microbiome may modulate oestrogen load which may be pertinent in obesity [84].

Obesity is often accompanied by hyperglycaemia [18], providing dividing cells with more fuel and affecting growth factor secretion [85, 86]. Insulin is released in response to increased blood glucose. Indeed, the hormone insulin and its structural relation, insulin-like growth factor 1 (IGF-1), have been linked to both carcinogenesis and obesity [87]. Insulin is produced in the pancreas in response to raised glucose levels, while IGF-1 is largely produced by hepatocytes in response to growth hormone, although it is made in many cells, including adipocytes [88]. Insulin levels rise chronically in obesity [51], and type 2 diabetes, which is characterised by insulin resistance and thus high circulating levels of insulin. Type 2 diabetes has been linked to an increased incidence of human obesity-related cancers including colorectal, hepatic, pancreatic, breast and endometrial [89]. Recent work on the *scrib* gene, which is involved in setting cell polarity, reveals that raised *Drosophila* insulin-like peptides systemically decrease tumour-suppressive cell competition and thus initiating tumorigenesis. Although this work was carried out in *Drosophila* [90], *scrib* has homologs in mice as well as humans, and appears to be a tumour suppressor in hepatocellular carcinoma in these organisms [91].

IGF-1 has also been linked to carcinogenesis [57–60]; however, in contrast to insulin, there is much debate surrounding circulating total IGF-1 levels and obesity in humans [57]. However, circulating total IGF-1 does not always reflect IGF-1 tissue levels, which are governed by the levels of IGF binding proteins (e.g. IGFBP-2) [58]. IGFBP-2 has been shown to decrease in obesity and hyperinsulinemia, potentially increasing the levels of free IGF-1 [62, 92]. Other studies looking at IGF binding proteins in obesity have not always shown consistent results. IGFBP-1 and IGFBP-3 may show no significant change in obesity [63], or an increase in IGFBP-3 [64]. Larger prospective studies are needed here. Interestingly, there is crosstalk between IGF-1 and oestrogen, for instance the IGF-1 promotor contains an oestrogen responsive element and IGF-1R is upregulated by oestrogen [93, 94]. In breast tissue, IGF-1 might operate as a paracrine signal, being produced in the stromal cells but promoting tumour growth in the epithelium as demonstrated in ex vivo models [95]. IGF-1’s close relative, IGF-2, may have a role in fat distribution and insulin resistance. IGF-2 is raised in circulation in human obesity and reduced after weight loss [61] and can increase cellular proliferation through the insulin receptor, at least in 3T3-like fibroblasts generated from mouse embryos nullizygous for IGF-1 and IGF-1R [96]. Furthermore, upregulation of IGF-2 occurs in a number of human malignancies, including breast, oesophageal and colorectal cancer [97]. The involvement of both insulin-like growth factors in the link between obesity and cancer remains to be clarified.

High blood levels of the cytokine IL-6 occur in patients with obesity [29] and in patients with hepatocarcinoma [98]. IL-6 is released by macrophages associated with adipose tissue, and stimulates cell growth in hepatocellular carcinoma mouse models through STAT3, ERK, and JNK activation and alterations in AKT and mTOR [99]. Another putative growth factor is TNFα which can cause phosphorylation of JNK and p38. TNFα production in immune cells is stimulated by leptin in humans [100] and this could be important in systemic inflammation (see ‘Avoiding immune destruction’ and ‘Tumour-promoting inflammation’).

Indeed other, less conventional, modulators can act as growth stimulators in patients with obesity. These include adipose-derived fatty acid binding protein (A-FABP). A-FABP promotes the migration and proliferation of breast cancer cell lines in vitro (MCF-7, E0771) and thus may be a novel factor driving proliferation in obesity-induced tumours. A-FABP is elevated in the serum of obese humans and has been shown to promote proliferation and invasiveness by enhancing the stemness of mammary tumours [70]. Members of the fibroblast growth factor family, fibroblast growth factors (FGF) 19, 21 and 23, are significantly changed in overweight and obese humans [53–55]. FGF23 is found in higher levels in overweight and obese individuals. It is a well-recognised growth factor in vitro for many cell lines (e.g. LNCaP, PC3), and FGF23 has been reported to be elevated in the serum of various cancer patients, including multiple myeloma [101]. The other members of the FGF family are discussed in ‘Reprogramming energy metabolism’. Another modulator is kynurenine, a tryptophan metabolite associated with excessive food intake. Kynurenine has been linked to both breast and colorectal cancers [102, 103] and acts as a major endogenous activator of the aryl hydrocarbon receptor (AhR) [104] which can increase adipocyte proliferation, and tumour progression. It is also noteworthy that AhR can be activated by lipid peroxidation products which can be elevated in obesity (see ‘Genome instability and mutation’).

Obesity can increase proliferation through a number of pathways, with perhaps leptin taking a central role. Further work is required to clarify the roles of other major protagonists, particularly looking at the nuanced role of oestrogen in different tumour types, thinking laterally about how to measure the true effects of IGF-1 in obesity and measuring markers of insulin signalling over time in human obesogenic tumour sites. It should also be noted that obesity can influence proliferation of immune cells (see ‘Tumour-promoting inflammation’) and proliferation with associated tumour growth affects local tissue invasion (not completely independent of ‘Activating invasion and metastasis’).

## Evading growth suppressors

In normal cells, control of growth represents a balance between growth-promoting compounds (growth factors) and growth suppressors. Excessive proliferation, as in cancer, can represent a hyperactivation of growth factor receptors, either by excess production of the growth factor itself (as outlined above) or mutation of proteins within signalling pathways. A further possibility is inhibition of growth suppressors and this is the focus of this section.

Adiponectin is generally believed to be a growth suppressor released by adipocytes, with both endocrine and paracrine actions. Adiponectin is known to increase insulin sensitivity, helping to regulate peripheral glucose and fatty acid metabolism [105]. Besides being a metabolic regulator, adiponectin has anti-inflammatory and anti-oxidant activity. Normally, adiponectin is present in the blood at relatively high levels (10 μg/ml), 1000 × higher than leptin and 10^6^ × higher than IL-6 or TNFα [34, 106]. In human obesity, circulating adiponectin falls by up to tenfold as visceral fat area increases [12] (Fig. 2). It seems paradoxical that adiponectin, which is produced by adipocytes, falls when the quantity of adipose tissue increases but this might be due to suppression by the paracrine actions of TNFα [107]. Low levels of circulating adiponectin are observed in humans with endometrial cancer and are associated with increased breast cancer risk after adjustment for BMI and age [108, 109]. Adiponectin suppresses cell proliferation in oestrogen receptor positive (MCF7) and negative (MDA-MB 231) breast cancer cells as well as hepatocellular (HepG2, Huh7) and endometrial (HEC-1-A, RL95-2) cancer cell lines [110–113] and its drop in obesity may contribute to cancer cells evading growth suppression. Adiponectin exerts its main actions through 7-transmembrane receptors (AdipoR1, AdipoR2) [114], ultimately inactivating the pro-proliferative mTOR [115] and also suppresses aromatase activity in adipocytes, lowering oestrogen production [111, 116]. However, controversies exist, with other work showing pro-proliferative effects of adiponectin in MCF-7 cells [117] and so perhaps further laboratory work should be undertaken to explore competing claims.

Several tumour suppressor proteins are reduced in obesity. Normally, TP53 is unstable in the cell, but it can be stabilised by phosphorylation by AMPK, which is activated by adiponectin [118]. Thus, it might be expected that TP53 concentration falls as adiponectin falls in obesity, and indeed, this fall appears to be exacerbated by leptin in cellular models (Swan-71) [119]. In contrast, some authors also claim that TP53 levels increase in obesity, exacerbating the release of inflammatory cytokines [120]. Whichever effect is dominant, a potential influence of TP53 on tumorigenesis in obesity cannot be ignored. Furthermore, polymorphisms of TP53 have been linked to obesity and lean body mass in mice and humans [121, 122]. Another tumour suppressor, PTEN, attenuates the PI3K-Akt pathway [123], the most commonly activated pathway in human cancers [124]. This tumour suppressing phosphatase causes the breakdown of PIP_3_, and switches off the Akt pathway. Adipocyte enhancer-binding protein 1 (AEBP1) rises in obesity, causing a drop in PTEN activity and consequent stimulation of adipocyte proliferation in mice [69] and this increase in adipocyte proliferation may lead to further crosstalk between adipocytes and tumour cells, for instance by leptin. Knockdown of AEBP1 reduces proliferation in various cell lines including colorectal, gastric and glioblastoma cell lines (HT-29, MGC803, XN0422, U87MG, U138MG), and high expression of AEBP1 has been linked with poor prognosis in colorectal and other malignancies [125].

Members of the TGFβ family are complex players in tumour development [126]. These are produced in macrophages and other lymphoid cells, and their levels, particularly TGFβ1, rise in obese adipose tissue in mouse models as well as in plasma in human obesity [35, 127]. TGFβ can act as a tumour suppressor in early cancer development [128]. However, TGFβ can also promote stemness and metastasis, and this effect might be more dominant in patients with obesity. Notably, leptin has been shown to increase protein levels of TGFβ1 in ER + and ER- breast cancer cells (MCF7, MCF10AT1) and a neutralising antibody against TGFβ1 blocks leptin-mediated actions [129]. Further work is needed to clarify the role of TGFβ in the different stages of cancer in obesity.

There may also be dietary effects on tumour suppression. High-fat diets cause inhibition of checkpoint proteins through lysine homocysteinylation of ataxia-telangiectasia and Rad3-related protein (ATR) in mouse colorectal cancer models [130]. This provides a mechanism by which cells can avoid cell-cycle arrest and apoptosis and exhibit accelerated proliferation. Diets with excess of protein can lead to overproduction of gut serine proteases (such as chymotrypsin) [16]. These may proteolyse another group of tumour suppressor proteins (DCC, neogenin), which interact with extracellular matrix proteins, the netrins. This effect has been demonstrated in neuroblastoma and colorectal cell lines (SH-SY5Y, CaCo-2) [131] but how far this applies to sites outside the gut in humans is unclear. Another serine protease, proprotein convertase subtilisin/kexin type 9 (PCSK9), has been shown to be raised in obese human serum [20] and also linked to melanoma metastasis in mice through its role in maintaining high circulating cholesterol levels, and likely activation of the TNFα pathway [132].

The drop in adiponectin might be the key factor in evading growth suppression, but this needs more work on a wider range of tumours as it may not be universal. The role of the TGFβ family most certainly needs further clarification and perhaps further work on the other tumour suppressor polymorphisms, like those related to TP53, might yield new pathways linking this hallmark with obesity. It should be noted that it is not always easy to separate the positive effects on growth suppression with negative effects on proliferation but we have taken classically defined growth suppressors, such as PTEN, as exemplars.

## Resisting cell death

Eukaryotic cells are programmed to die, either after a particular number of divisions in maintenance of organs or in response to a great variety of stresses; hence, avoiding death is a key feature of cancer cells. Leptin, a key player in growth stimulation in obesity, inhibits apoptosis, largely by increasing the expression of the anti-apoptotic proteins B-cell lymphoma 2 (Bcl-2) and survivin. This phenomenon has been widely studied in cell lines (e.g. HepG2, MCF-7 and OVCAR-3) [133, 134]. IGF-1 also opposes apoptosis through the Bcl system in cellular models, possibly by decreasing (proapoptotic) TP53 [135]. Obesity can also influence apoptosis by upregulating collagen VI, which is produced during adipose tissue fibrosis and its cleavage product, endotrophin, has an anti-apoptotic effect in murine mammary tumours [136].

Cell death outside the tumour itself may promote resistance to death amongst tumour cells. In obesity, adipocyte hypertrophy is common and adipocytes more frequently undergo apoptosis [137] or necrosis [138]. This process attracts macrophages and the dying adipocytes and their surrounding ‘crown-like’ structures are typical of various fat deposits in human obesity, including subcutaneous and visceral fat [138, 139]. These dying cells release cytokines, which can drive cell division and further inflammation. IL-6 in particular has anti-apoptotic actions, through upregulation of Mcl-1 (a member of the Bcl-2 family) in a number of cancer cell lines [140, 141] (e.g. C33A, AGS), including prevention of apoptosis in hepatoma cells under the influence of TGFβ [142]. Furthermore in obesity, hypoxia is said to occur in adipose tissue secondary to a number of mechanisms, including cardiac output not keeping pace with adipose expansion and aberrantly reduced postprandial blood flow [143]. However, this is controversial in at least some adipose tissue deposits [144]. If present, hypoxia can drive tumorigenesis in number of ways [145, 146], including via hypoxia inducible factor 1α (HIF1α) and leading to the production of VEGF-A [147] which has anti-apoptotic actions in adenocarcinoma models [148]. Indeed, hypoxia can have local and systemic effects and thus can directly influence hypoxic cells or others further afield. Such effects are further discussed in ‘Inducing angiogenesis’ and ‘Reprogramming energy metabolism’.

Another route to programmed cell death is pyroptosis, which differs from apoptosis in (a) involvement of the inflammasome, (b) dependence on caspase 1 activity (rather than caspases 3,6,8 which are activated in apoptosis) and (c) being a pro-inflammatory process, releasing the cytokines IL-1β and IL-18 [149]. Pyroptosis is normally involved in defence against invading bacteria: the cell membrane develops pores, the cytoplasm swells and the cell eventually ruptures, removing intracellular niches for growth of these bacteria. Pyroptosis has been shown to be upregulated in adipose tissue and is a source of adipocyte death in murine obesity [150] and this may be linked to the formation of the ‘crown-like’ structures, and consequent chronic inflammation. Interestingly, the anti-diabetic drug metformin has been shown to induce pyroptosis in oesophageal cancer cell lines (KYSE510, KYSE140) and so, might be of benefit in this obesity-related cancer in the clinic [151].

Of note, several of the factors influencing apoptosis in obesity may have pro- or anti-apoptotic effects dependent on context. Indeed, a number of competing processes seem to be involved. For example, kynurenine and its metabolites can induce apoptosis of both NK cells and T-cells, (potentially allowing cancer cells to avoid immune destruction) while inhibiting apoptosis in colorectal cancer cells (HT-29) [152–154]. Also, exosomal transfer of small non-coding RNAs from adipocytes may regulate biological processes through post-transcriptional gene silencing. miRNAs, particularly miR-21, from cancer-associated adipocytes, can inhibit apoptosis in ovarian cancer cell lines (SKOV3, OVCA432) by targeting apoptotic protease-activating factor 1 (APAF-1) [155].

Overall the effects of obesity on the mechanisms of avoiding cell death are mixed, with some factors dominating in some scenarios, and other effects less clearly understood. Perhaps, pro-death signals may simply be drowned out in some circumstances, when one considers the complex interaction between potentially anti-apoptotic factors (such as VEGF-A and IL-6) and the soup of growth factors circulating in obesity.

## Enabling replicative immortality

While normal cells will undergo a finite number of divisions before senescence, cancer cells acquire unlimited replicative potential [10]. Multiple lines of evidence indicate that telomeres protecting the ends of chromosomes are centrally involved in the capability for unlimited proliferation. Telomeric repeats shorten with each division, finally exposing non-telomeric DNA, precipitating end-to-end chromosomal fusions; causing unstable dicentric chromosomes, and limiting the ability to generate viable cells. Reactivation of telomerase has been identified as a major route by which cancer cells prevent telomeric erosion [156] generating longer telomeres and promoting extended proliferation/ immortality.

The influence of obesity on this hallmark is contentious, although some adipokines promote telomerase activity in specific cases. Leptin upregulates telomerase activity as well as the mRNA and protein levels of the catalytic subunit of reverse transcriptase of human telomerase (hTERT) in breast cancer cells (MCF-7) [157]. Plasma leptin levels are correlated with tumour hTERT levels in human breast cancer (*r* = 0.484, *p* < 0.01) [158] and leptin mRNA in human hepatocellular carcinoma is also strongly correlated with hTERT (*r* = 0.79, *p* < 0.01) [159]. Resistin, another raised adipokine in obesity [14], has also been shown to increase the expression of hTERT in gastric cancer cells (AGS) [160]. IGF-1 has also been implicated in telomere lengthening, although conflicts in the literature exist when contrasting model systems [161, 162]. It has also been claimed that the tumour suppressor TP53, which may be lowered in obesity (see ‘Evading growth suppressors’), downregulates the expression of hTERT in a cervical cancer cell line (SiHa) [163] in addition to its proapoptotic effects. However, in the main, telomeres appear to be shorter in patients with obesity [164], perhaps partially due to the influence of insulin [165], and shorter telomeres predispose cells to ‘Genome instability and mutation’.

It is known that in some cancers, there are alternative methods of telomere lengthening operative during the multi-stage carcinogenic process. One cause of alternative telomere lengthening (ALT) is aberrant DNA damage repair, and altered DNA damage repair pathways have been reported in obesity [166]. However at present, little appears to be known about ALT in obesogenic carcinogenesis.

At present, the link between replicative immortality and the obese state is not well-established. Leptin effects appear the most studied but literature is sparse with little published on other adipokines. ALT in obesity seems an inviting avenue for further work.

## Inducing angiogenesis

As tumours grow, they must generate neovasculature or co-opt pre-existing vessels. Adipocytes alter their adipokine profile in obesity, producing a plethora of pro-angiogenic factors including leptin, ceruloplasmin, angiopoietin-like proteins (e.g. ANGPTL2), hepatocyte growth factor (HGF) and proteins of the vascular endothelial growth factor (VEGF) family, including placental growth factor (PlGF), and hence promote an environment conducive to tumour growth [66].

The primary stimulant for angiogenesis is VEGF. Binding of VEGF-A to its receptor activates the intracellular VEGF cascade, ultimately stimulating endothelial cell proliferation, migration and vascular permeability [167]. Leptin has been reported to increase secretion of VEGF-A in murine adipose tissue in mice [168] and VEGF-C in human chondrosarcoma cells (JJ012) [169]. Indeed, elevated serum VEGF-A, VEGF-C and VEGF-D have been described in obese humans [66]. Leptin also increases receptor expression (VEGFR-2) in ﻿mammary tumours in mice [170] and its transphosphorylation in cellular models (HUVEC) [171]. Alongside this, leptin has been described as a vasoactive hormone. Leptin has been demonstrated in Wistar rats to cause vasodilation through nitric oxide release [172] and inhibition of angiotensin II [173].

Another pro-angiogenic factor, tissue factor (TF), is overexpressed in adipose tissue in obese mouse models [49], and plasma TF is reduced by weight loss in obese humans [174]. It has been proposed that increased TF expression by endothelial cells enhances angiogenesis, and increased TF expression in tumour cells (Meth-A) and human pancreatic tumours was associated with increased VEGF expression [175, 176].

Angiopoietin-like proteins (ANGPTLs) are other angiogenic and metabolism-modifying factors that can be upregulated in obesity. Of particular note is ANGPTL2 which is abundantly expressed by adipose tissue and whose serum levels are positively correlated with body mass index [71]. ANGPTL2 has been linked with obesity-related cancers in humans, for instance colorectal cancer [177] and has been shown to increase angiogenesis by increasing VEGF-A and Ang II in osteosarcoma mouse models [178]. Also ANGPTL2 has been shown to induce vasculogenesis directly in endothelial colony forming cells, activating c-Jun NH2-terminal kinase (JNK) and increasing membrane type 1 matrix metalloproteinase (MT1-MMP) [179].

TGFβ and TNFα are elevated in the adipose tissue of obese mice and may have paracrine effects on angiogenesis. If participants in the TGFβ signalling pathway increase, clinical conditions with excess angiogenesis can manifest, such as hereditary haemorrhagic telangiectasia (HHT) and Osler-Rendu-Weber syndrome, suggesting that TGFβ has a pro-angiogenic action [180]. TNFα induces and modulates angiogenesis in a number of ways, including induction of VEGF-D [181]. It is unclear, however, how far these effects are dependent on intermediate cytokine production, e.g. IL-8, which is elevated in the serum of obese humans [30].

The reduced adipocyte production of adiponectin in obesity may influence angiogenesis. Adiponectin has been shown to inhibit angiogenesis through several pathways, including its effects on the rho kinase/IFN-inducible protein 10, matrix metalloproteinase 9 and the MAPK and cAMP-PKA pathways [182, 183], and so its reduction may promote angiogenesis. Adipose tissue also synthesises pro-angiogenic hepatocyte growth factor (HGF). HGF is elevated in patients with obesity [56] is linked to hepatocellular carcinoma [184].

Other potentially unexpected factors might also have a role in promoting angiogenesis. Ceruloplasmin, a serum ferroxidase involved in copper transport, is raised in patients with obesity [74, 185], and induces angiogenesis probably by stimulating VEGF-A production via the HIF1α signalling pathway. Also, the aforementioned AhR can be activated in by kynurenine. Like HIF1α, when activated, the AhR moves to the nucleus and activates genes associated with hypoxia, including VEGF-A [186].

Hypoxia itself likely links many of these angiogenic mediators and is a well-established and key regulatory factor in tumour growth [145, 146]. In obesity, the HIF1α pathway can be activated [187]. This markedly increases the production of adipocytokines [188] including leptin, VEGF-A and HGF.

Obesity seems to have a marked impact on angiogenesis, with many factors ultimately acting through VEGF-A. Tissue hypoxia is a major stimulant of angiogenesis and may influence proceedings whether it occurs in subcutaneous deposits of adipose tissue or in organs where ectopic fat deposition occurs (e.g. pancreas). More investigation is needed as to the roles of ANGPTLs and the effects of ceruloplasmin on angiogenesis in human obesity-related tumours.

## Activating invasion and metastasis

An important pathological feature of cancer cells is their ability to invade surrounding tissue, and also metastasise to distant sites. A familiar pair of adipokines, leptin and adiponectin, influence invasion and metastasis. To allow this process, cells tend to undergo a dedifferentiation process which some have termed epithelial-mesenchymal transition (EMT) and the Hedgehog developmental gene complex is involved in this process. Leptin activates the Hedgehog developmental complex [189], and EMT in breast cancer cell lines (MCF-7, SK-BR-3) [190]. Leptin also stimulates the production of IL-6 and TGFβ which have paracrine activity reinforcing EMT [191], while promoting secretion of extracellular matrix remodellers, such as matrix metalloprotease 2 (MMP2) in mammary cells (MCF10A) [68]. Reducing leptin availability to implanted ER + breast cancer cells (MCF7) significantly decreased the number of metastatic lesions in mice [192]. Resistin, also raised in obesity, induced EMT in breast cancer and mammary epithelial cells (MCF7, MDA-MB-231, MCF-10A) [193]. Conversely, adiponectin can reduce leptin-induced metastasis typically by activating AMPK [194]. Thus, the reduction of adiponectin seen in obesity can favour metastasis.

Hyperinsulinemia is commonly associated with obesity. Insulin can increase the migration ability of colorectal cancer cells (HCT-116) via PI3K/Akt and MAPK [195], while downregulation of the insulin receptor reduces lung metastasis in a mouse model with the highly metastatic breast cancer cell line, LCC6 [196]. Furthermore, hyperinsulinemia appears to influence response to cancer treatments. The non-small cell lung cancer cell line (HCC4006) harbours a EGFR mutation normally rendering it sensitive to the tyrosine kinase inhibitor gefitinib. Co-treating cells with gefitinib and insulin increased cellular proliferation and survival. This treatment resistance was attributed to insulin activating the PI3K/AKT pathway [197] and this perhaps plays a role in treatment failure in patients with obesity. It does also suggest that insulin provides a PI3K-activating stimulus without having a specific mutation in this important pathway.

Importantly, invasion, migration and the formation of metastases are complex processes involving tumour and other cells. Gene expression profiling of obese/overweight human periprostatic adipose tissue highlighted differences likely to cause a local environment favourable to prostate cancer progression [198]. Furthermore, adipocytes in proximity to tumour cells promote invasiveness in a number of different cell lines (e.g. Pt-93, SW480) and mouse models [199, 200].

In breast cancer, adipose-derived stromal cells (ASCs) can release fatty acids to feed tumour cells and release influential miRNAs and cytokines. Indeed, IL-6, IL-8 and leptin secreted by ASCs promote invasion by breast cancer cells (MCF7, MDA-MB-231) [201, 202]. ASCs also show increased expression of IGF-1 in obesity, enhancing local invasion in mice [203] possibly through its effect on anoikis (elaborated later in this section). ASCs influence the extracellular matrix, depositing denser and stiffer matrices in obesity mouse models. This stimulates growth and invasion through local increased mechanosignaling and increased YAP/TAZ transcription factor activity and potentially further afield through increased secretion of stromal cell–derived factor 1 (SDF-1) [75]. Adipocytes also produce a number of other metastatic-enabling compounds including MMPs, β-hydroxybutyrate, plasminogen activator inhibitor-1 (PAI-1), collagen VI, TNFα and IL-1β [28, 48, 67, 204, 205]. And, A-FABP from omental adipocytes appears to promote ovarian cancer metastasis in model systems (discussed further in ‘Reprogramming energy metabolism’) [206], while CCL7 from periprostatic adipocytes increases migration of tumour cells (PC-3, Du-145) *in vitro* [26]*.* This local crosstalk between tumour cells, adipocytes and their related cells appears key.

Adipose tissue also might have a role at a distance. Adipose tissue is a major source of circulating exosomal miRNAs, which can control gene expression in distant tissues, at least in mice [207]. These have been proposed to drive tumour progression, invasion and metastasis, through inducing fatty acid oxidation in melanocytes, Wnt signalling in mammary tumours and cell cycle progression in hepatocarcinoma [208–210]. In adipocyte/hepatic cell cocultures, an increase in the free miRNA miR-27a was linked to tumour development [73].

The invasion route for transformed cells is enhanced through angiogenesis and lymphangiogenesis. ANGPTL2, for example, is linked with promoting metastasis in human lung cancer and in mouse breast to lung metastatic models [211]. As discussed in the previous section, other factors that increase angiogenesis are present at higher levels in obesity. Once detached from the parent tissue, normal cells typically self-destruct (anoikis) [212] but cancer cells can develop anoikis resistance. Triggering of anoikis involves inhibition of mTOR via AMPK. In breast cancer cells (MCF7, ZR-75, EFM-19), anoikis has been shown to be opposed by IGF-1 (binding to IGF1R) [213]. Since AMPK activity is reduced in obesity, at least in human obese visceral fat [214], and IGF-1 may locally rise, obesity may oppose anoikis and promote metastasis. Indeed IGF-1 has been linked to several stages of invasion and metastasis [215].

**Fig. 3:**
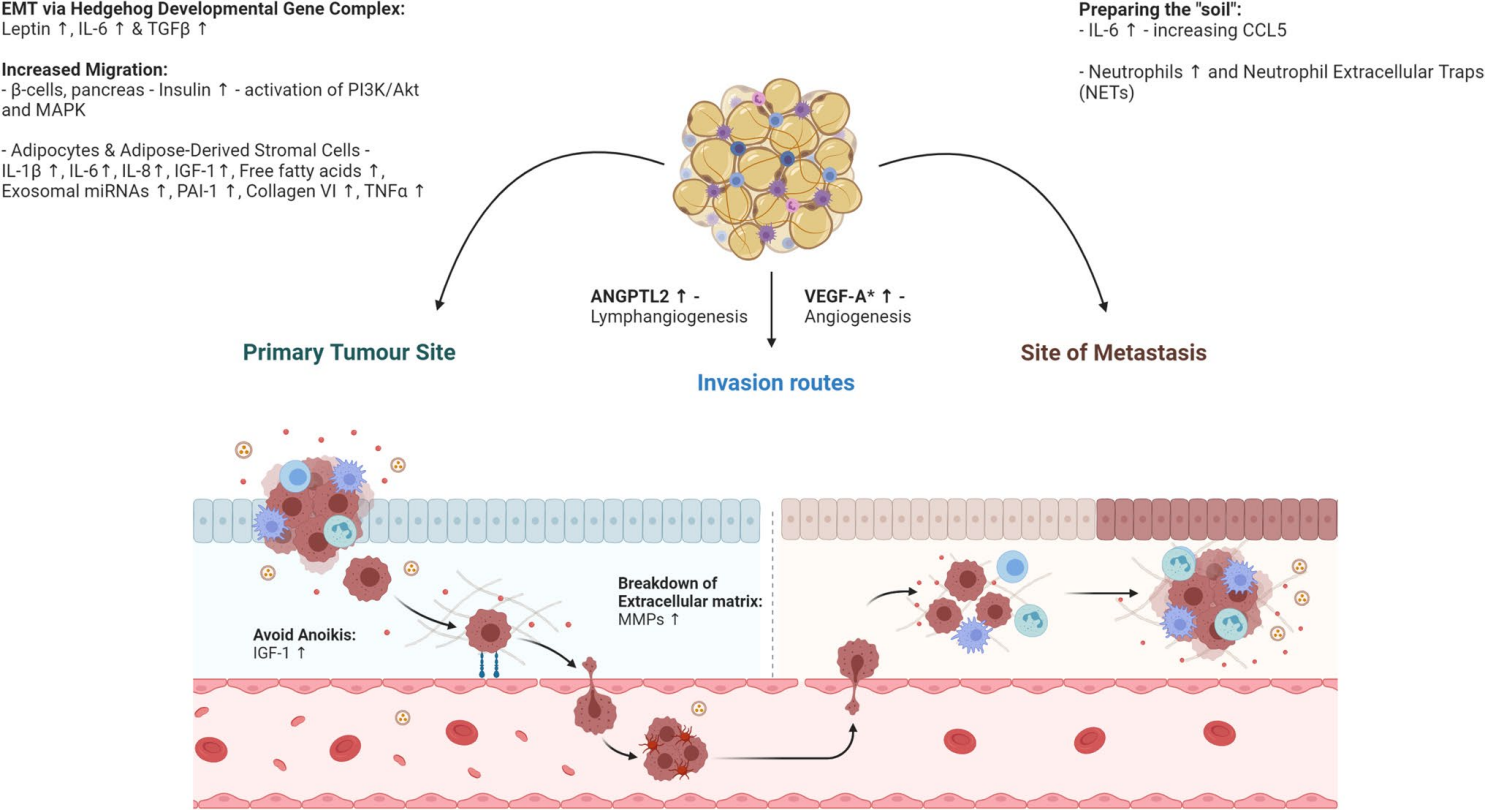
**Major factors that can promote cellular invasion and metastasis in obesity.** Migration, invasion and metastasis are influenced by a number of different factors in obesity. At the primary tumour site, leptin, IL-6 and TGFβ can promote epithelial-mesenchymal transition (EMT). Combining this with systemic hyperinsulinemia, exosomal influences and cross-feeding by nearby adipocytes cellular migration can be encouraged. Cells escape through the blood and lymphatic vessels, increased in number by elevated factors such as VEGF-A and ANGPTL2 (other factors reviewed in the ‘Inducing angiogenesis’ section). Furthermore, the physiological state of obesity acts to prime distant niches for metastasis through various mechanisms, for instance the induction of CCL5 expression

Obesity can also affect the target ‘soil’ of the metastatic cells. IL-6 has been linked with preparing metastatic niches by inducing CCL5 expression in lymphatic endothelial cells within pre-metastatic sites and creating chemotactic gradients to recruit CCR5-positive cancer cells into the lymph nodes and lungs in model systems of triple-negative breast cancer metastasis [25]. Obesity-associated lung neutrophilia appears to enhance breast cancer to lung metastases in mouse models [216]. Neutrophils also produce neutrophil extracellular traps (NETs), web-like structures of chromatin, which are pro-inflammatory. Increased plasmatic NETs have been seen in patients with morbid obesity [43]. NETs have been linked with induction of metastasis and promoting tumour growth, particularly in breast cancer models [217, 218]. A summary of how obesity can affect invasion and metastasis can be seen in Fig. 3.

It appears the obese state can prepare cells for all aspects of the metastatic process. This includes stimulating cell proliferation and de-differentiation, through release of tumour cells from tissues (including avoidance of anoikis), providing vessels for their dispersal and providing a final niche for their growth. As obesity is related to aggressive disease, metastasis and short survival in a number of cancer types, we suggest prioritisation of efforts to understand metastatic niche preparation in obesity will perhaps yield the greatest benefit to patients.

## An enabling characteristic: tumour-promoting inflammation

Obesity is associated with a chronic inflammatory state, in which immune cells accumulate in the adipose tissue and secrete cytokines. And, inflammation is widely accepted to be associated with tumour development and progression through a number of mechanisms [219].

Indeed, inflammation in tissues reflects a balance between anti-inflammatory and pro-inflammatory factors. In obesity, adipose tissue expands and the population shifts towards Th1 cells, CD8^+^ T cells and pro-inflammatory macrophages, while some T_reg_ and Th2 cell numbers decline [38, 44–47]. Notably, the macrophage population of adipose tissue can rise to 40–50% of total cells, compared to < 10% in lean adipose tissue [220]. Perhaps this is driven in part by monocyte chemotactic protein 1 (MCP-1), which is raised in human obesity [33] and has been shown to cause macrophage proliferation in adipose tissue in mouse models. Mouse models of obesity have also shown that communication between adipose tissue and bone marrow increases the production of myeloid cells [221]. The characteristics of adipose tissue macrophages are an area of much debate and are evolving. Currently, it is generally believed that pro-inflammatory macrophages (originally felt to be classical M1, but other data suggests a different pro-inflammatory phenotype [222]) are stimulated by leptin, to produce pro-inflammatory cytokines such as IL-6 [223]. Also, high levels of PlGF are associated with obesity in breast and pancreatic cancer patients [224] and raised levels of PlGF are seen in obese children and adolescents without cancer [65]. PlGF may also help shift macrophages into a pro-inflammatory state. TNFα and IFNγ are produced and the balance of the lymphoid population alters [27, 225]. TNFα promotes the production of cytokines such as IL-6 within adipose tissue [226], following its proteolytic release from the membrane. Eosinophils decrease in number in obesity, at least in mice [39]. These effects may influence the lymphoid populations in organs/tissues that tend to accumulate fat in obesity, such as the pancreas, breast and liver.

Recent work in triple-negative breast cancer patients and mouse models perhaps highlights the importance of a difference in lymphoid and myeloid populations. These studies investigated lean and overweight/obese patients, both with > 30% stromal tumour-infiltrating lymphocytes (TILs). They showed that lean patients have a higher rate of a complete pathological response to neoadjuvant chemotherapy (73.1%) than overweight/obese patients with the same total proportion of TILs (44.7%) [227]. This presumably reflects a different make-up of the TILs population. Alternatively, this effect could reflect different levels of T-cell exhaustion as discussed in the next section. As regards myeloid cells, an important player here might be metabolically activated adipose tissue macrophages (MMes). MMes, driven by saturated fatty acids (e.g. palmitate) released by insulin-resistant adipocytes, accumulate in mammary fat of obese mice and humans and promote stemness within the tumour via IL-6 [42]. Also tumour-infiltrating myeloid cells showed an activated NLRC4 inflammasome in breast cancer mouse models and these can lead to crosstalk with adipocytes to promote angiogenesis and disease progression [228].

**Fig. 4:**
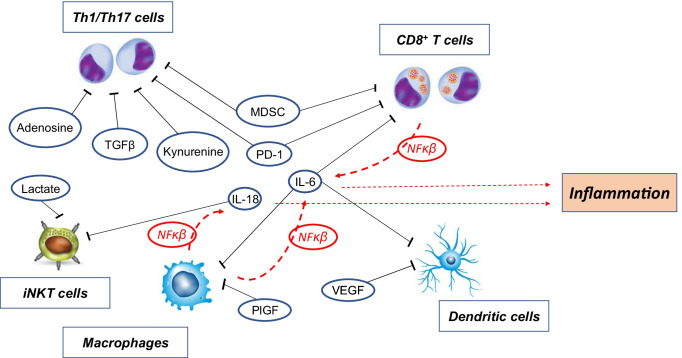
**Notable effects of obesity on immunosuppression and inflammation.** Cells relating to immune surveillance of cancer, raised in obese adipose tissue, are shown (Th1/Th17 and CD8 + T cells, dendritic cells, pro-inflammatory macrophages and iNKT cells). Suppression is shown as black blunted lines. Circulating cytokines, IL-6 (acts on dendritic, CD8 + T cells and macrophages) and IL-18 (acts on iNKT cells), are major mediators of immune suppression, but other growth factors/cells can suppress immune responses in dendritic cells (VEGF), macrophages (PlGF), Th1/Th17 cells (TGFβ, MDSC) and CD8 + T cells (MDSC). Small molecule effectors may also be involved-adenosine and kynurenine dampen the immune response in Th1/Th17 cells while lactate suppresses actions of iNKT cells. Simultaneously these cells, notably the pro-inflammatory macrophages and CD8 + T cells, release cytokines IL-6 and IL-18 (red arrows), resulting in a complex feedback system. They are stimulated to do so by extracellular mediators such as fatty acids (raised in obesity), DAMPS (from dying adipocytes in ‘crown-like’ structures), IL-1β and TNFα, via NF-κB, the predominant intracellular coordinator of inflammation

A key orchestrator in the induction of inflammation is the intracellular mediator, NF-κB, which, when activated, moves to the nucleus to drive the expression of inflammatory mediators, such as IL-6 and IL-18 (Fig. 4). Its accessory protein, IKK-β, can sense nutrient levels, notably fatty acids [229], leading to the activation of NF-κB and consequent production of inflammatory cytokines in diet-induced obesity. This is parallel to NF-κB activation through Toll-like receptors (TLR), which are discussed below. Indeed, active NF-κB has been described to play a major part in the onset of liver and intestinal carcinogenesis [230]. Opposing inflammation are the inherent anti-inflammatory actions of the Wnt/β-catenin system. In adipocyte hyperplasia, this system is downregulated, leading to an increase in inflammation. These changes are also associated with changes in the T cell population, away from important subtypes of T_reg_ cells [231].

Furthermore, in obesity, the adipocytes themselves can necrose. The dead cells release lipids, nucleic acids, damage-associated molecular patterns (DAMPs) etc., all of which attract macrophages. These form ‘crown-like’ structures around the dying adipocytes [232]. Activation of these macrophage pattern receptors activates inflammasomes [233] generating the inflammatory IL-1β, and more macrophages are attracted by endotrophin produced by adipocytes [234].

The microbiome is notably different in patients with obesity [235]. This may affect carcinogenesis through local and systemic inflammation, possibly by allowing the leakage of lipopolysaccharide (LPS), which activates TLR4 and stimulates pro-tumorigenic inflammation [230]. Indeed, plasma LPS levels in both humans and rodents with obesity and insulin resistance are approximately doubled compared with those in healthy controls [24, 236]. In addition, a healthy microbiome produces short-chain fatty acids, such as butyrate, which have anti-inflammatory effects, and it is possible that the altered microbiome in obesity might be less effective in opposing inflammation due to decreased production of short-chain fatty acids. Another putative mechanism is an effect of the microbiome in obesity on bile acid metabolism. Deoxycholic acid, a secondary bile acid, increases senescence-associated secretory phenotype (SASP) and thus the pro-inflammatory IL-1β and leads to hepatocellular carcinoma in mice [21]. At the opposite end of the alimentary tract, the oral microbiome and inflammation of the oral cavity owing to bacteria (periodontitis) have been linked with obesity and also to cancer. Recent work has highlighted a link between periodontal disease, oesophageal adenocarcinoma and gastric carcinoma [237]. Antibodies to and carriage of various oral pathogens (e.g. *Porphyromonas gingivalis*) are related to an up to two-fold increased risk of pancreatic cancer [238], indicating some systemic involvement. However, prospective and interventional studies involving the microbiome are needed.

Excess body fat and its influence on the immune system are undeniable with both myeloid and lymphoid cells affected. IL-6 appears a key cytokine in the chronic inflammatory state and thus likely in obese tumorigenesis. Controversies still exist surrounding exact macrophage profiles in obesity and the true extent of the influence of microbiota, which single-cell sequencing and interventional studies should help clarify.

## An emerging hallmark: avoiding immune destruction

As we have seen, in obesity, adipose tissue shows the characteristics of chronic inflammation, with the accumulation of some types of immune cells including certain T cells and macrophages which release a variety of cytokines. As such, it might be expected to be primed for immune response against tumours. However, this does not seem to be the case [239].

White adipose tissue (WAT), particularly visceral WAT, is readily colonised by immune cells, particularly macrophages, T_reg_ cells, eosinophils, invariant natural killer (iNKT) T cells and mucosal-associated invariant T (MAIT) cells. In lean subjects, the population of immune cells (particularly macrophages and iNKT cells) seem to maintain ‘immune surveillance’; they are able to detect and kill potential tumour cells while producing cytokines (e.g. IL-10) which attract and maintain this population. CD4^+^ and CD8^+^ cells are also present and involved in killing cancer cells [240]. Since adipose tissue shows an increase in lymphoid content in obesity, one might expect it to be more efficient in its immune surveillance. In fact, this surveillance system appears compromised [241]. There are changes in the immune cell content in obese adipose tissue in humans and mice [242]. T_reg_, iNKT, MAIT and eosinophil numbers decline, while the macrophage population increases and shifts to pro-inflammatory state; exhausted memory B cell populations rise and IL-6 becomes the dominant cytokine produced [36, 37, 40, 232, 243].

Obesity has been shown to compromise mechanisms regulating T-cell generation by inducing premature thymic involution in mouse models. Diet-induced obesity significantly increased apoptosis of developing T-cell populations and reduced T-cell diversity [244]. Raised circulating fatty acid concentration in the tumour microenvironment might in some cases also reduce the numbers of tumour-infiltrating CD8^+^ T cells by inducing iron-dependent cell death (ferroptosis) via CD36 [245]. As well as reducing numbers of antitumour cells, it seems that the normal immunosurveillance role of Th1 cells are blunted by the presence of cytokines secreted into the extracellular milieu shifting the balance away from cytotoxic responses towards inflammatory ones [246]. Type 2 diabetes in mice and humans, which is also associated with obesity, has been shown to reduce the multifunctionality of peripheral CD8^+^ T cells in response to both non-specific and antigen-specific activation [247]. In obesity, T cells become ‘exhausted’ leading to a lack of function (‘inflammaging’) [244]. This phenomenon has been shown across multiple species, tissues and tumour models and is driven in part by leptin signalling [248]. There is an increased expression of the programmed death receptor (PD-1) on the surface of CD8^+^ T cells, and this binds to PDL-1 on the surface of tumour cells, preventing the T cells killing their targets [248]. The influence of obesity on the PD-1/PDL-1 axis may explain its unexpected benefits on the efficacy of immunotherapy in cancer patients [249], although other work has not confirmed such benefits and larger more in-depth prospective trials are needed to clarify any potential benefit [250].

Somewhat paradoxically, the hormone most affected by obesity and linked to tumour growth (above) seems to promote antitumour activities by the immune system [251]. Leptin has been shown to influence the development of NK cells [252], dendritic cells (involved in antigen presentation) [253], Th1 lymphocytes [254] and eosinophils (involved in the adaptive and innate immune responses) [255] while decreasing the level of T_reg_ cells [251]. Leptin also stimulates the release of IL-6, IL-1β and TNFα (particularly from adipose tissue macrophages) [256] which, together with MCP-1 (also released in inflammation), seem to attract myeloid derived suppressor cells (MDSC) which can suppress important CD8^+^ T cell-mediated antitumor immunity [41, 257–259]. So there seems to be a balance between leptin promoting immunosurveillance, while at the same time causing the accumulation of MDSCs which oppose it, although the majority of the work above was in model systems.

Other factors in the tumour microenvironment have been implicated in immune suppression in obesity. IL-18 is raised in obesity (both in tissues and in the circulation) [32], and while it directly promotes cell proliferation in stomach cancer and melanoma, it can also drive immune evasion by decreasing the expression of CD70 which normally increases the activity of antitumour NK cells [260]. Furthermore, human and mouse NK cells can become ‘paralysed’ in obesity showing increased lipid accumulation, impaired lytic machinery mobilisation and loss of cytotoxicity against tumour cells [261].

AhR is expressed at high levels in several tumours, and promotes immune tolerance by increasing the proportion of T_reg_ cells (immunotolerant) at the expense of CD8^+^ T cells at least in human glioma [104]. AhR is activated by endogenous ligands such as kynurenine, whose levels rise in human obesity [19] and which is itself metabolised into a number of products, particularly 3-HAA, which suppresses the pro-inflammatory mouse Th1 and Th17 cells [262]. Furthermore, macrophages, attracted into the tumour microenvironment in response to MCP-1, produce TGFβ and IL-10 which suppress the cytotoxic activities of Th1 cells [263]. MCP-1, TGFβ and IL-10 have all been described as elevated in the serum of patients with obesity [31, 33, 35]. The production of arginase and of indoleamine dioxygenase is upregulated, leading to depletion of arginine and tryptophan that immune cells need for proliferation. Increasing lactate and adenosine in the tumour microenvironment may also promote immunosuppression [264]. Furthermore, high-fat diets in mice paradoxically appear to starve CD8^+^ T cells of lipids required for their proliferation. This has been attributed to reprogramming of energy metabolism towards fatty acid catabolism in adjacent tumour cells, through a decrease in proline hydroxylase-3 (well-known for its regulatory impact on HIF1α) [265, 266]. CD8^+^ T cells may also undergo metabolic reprogramming. In breast cancer mouse models, obesity activates the leptin-STAT3 axis causing increased fatty acid oxidation and a reduction in CD8^+^ T cell antitumor function [267]. And in sarcoma mouse models, it has been shown nutrient competition, particularly glucose, between tumours and T cells is a distinct mechanism, which can lead to T cell hyporesponsiveness [267, 268]. Other work in mice has shown that the cholesterol metabolite, 27-hydroxycholesterol, which is thought to be increased in obesity [15], increases the number of polymorphonuclear neutrophils and γδ-T cells at distant metastatic sites in breast cancer models and this might enhance metastasis by polarising towards an immune-suppressive subtype [269].

Obesity appears to allow tumour cells to evade destruction mainly through its diverse effects on T-cells. Reduction in T-cell production and diversity, coupled with exhaustion, appear potentially important, but perhaps provide an ‘Achilles heel’ through PD-1 targeting. The role of AhR is intriguing and is worthy of further investigation.

## An enabling characteristic: genome instability and mutation

Excess body fat has been linked with locally increased production of reactive oxygen species (ROS), mainly generated in mitochondria [23, 270]. ROS species damage DNA, and mutations often arise from attempts to repair this damage. Oxygen radical formation varies, with circulating fuels (increased glucose, fatty acids) and/or hormones playing a role. Possible mechanisms involve activation of NADPH oxidase (which transfers electrons from NADPH to molecular oxygen) and protein kinase C [271]. For example, abnormally higher levels of circulating insulin, due to insulin resistance in obesity, can cause DNA damage by increasing the translocation of Akt into mitochondria and its activation of NADPH oxidase. Oxidative stress can lead to an increase of lipid peroxides, alkoxyl radicals, aldehydes and ketones. Elevated lipid peroxide levels (estimated by plasma malondialdehyde and 4-hydroxyalkenal levels) have been repeatedly demonstrated to accompany fat accumulation in humans [22, 272]. Aldehydes can lead to genomic instability through glycation which activates receptor advanced glycation end product (RAGE) receptors and their downstream pathways, which include the NFκβ pathway [270]. Indeed, the aldehyde methylglyoxal has been shown to cause DNA modification and strand breaks [273]. Free radicals can also cause damage to the hexameric repeats within telomeres, accelerating telomere loss during the cell cycle.

While we noted above that longer telomeres lead to an increased cancer risk, so too do shorter telomeres and in pre-malignant cells, telomeres can be extensively eroded [274]. Telomere erosion leads to chromosomal abnormalities including fusion, translocations and breakages, all of which contribute to genome instability. There are also secondary effects on genome stability. The hormonal effects of obesity, including raised oestrogen, and decreased steroid binding globulin [275], drive proliferation (see ‘Sustaining proliferative signaling’) and increase the chance of replication error. In addition, oestrogen and its metabolites, which can be produced peripherally by cytochrome P450 isozymes (mainly CYP1A1), can cause free radicals and DNA adducts with similar consequences [166]. Several DNA repair pathways are potentially altered in the obese state. Indeed, as discussed in the ‘Evading growth suppressors’, ATR can be inactivated [130], leading to inability to repair double strand breaks and aberrant replication fork structures [276]. Reliance on different DNA repair pathways may explain varying oncogenic susceptibility between tissues, dependent on their dominant pathway for DNA repair [277].

DNA damage has long been associated with carcinogenesis, and obesity has been shown to influence the cause and repair of DNA damage. With specific pathways affected, e.g. lipid peroxidation, it is possible that the physiological state of obesity provides a selective pressure for certain mutations within tumours. The precise effectors of the selective pressure are currently uncertain.

## An emerging hallmark: reprogramming energy metabolism

There must be considerable metabolic change in tumour cells, to provide both the energy and the raw materials for cell growth and proliferation. While this might be considered simply a consequence of cells adjusting to growth modification in cancer, the discovery of oncogenic metabolites associated with the tricarboxylic acid cycle and the ability to modify pathways as a route to cancer therapy show the importance of altered metabolism as a cancer hallmark [278]. Some go so far as to classify cancer as a ‘metabolic disease’ [279], and certainly the interplay between cell growth and proliferation, and energy metabolism discussed below highlight the requirement for tight integration between cell energy metabolism and tumour growth.

In obesity, there is an abundance of circulating glucose and thus tumours are provided with necessary fuel. Abundant glucose supply is associated with a stimulation of glycolysis. Expression of the unusual isoforms of glycolytic enzymes, PKM2, LDHA and GLUT1,3 is common in tumour cells. Mitochondrial respiration may also be depressed by leptin as shown in colon cancer cells (HCT116) [280], possibly indicating a shift to a glycolytic phenotype. Indeed, there is increasing evidence that tumour metabolism can switch between glycolytic and oxidative metabolism depending on tissue of origin, cancer progression and malignancy [281]. This may be dependent on the cancer type. One study has claimed that cell lines from obesity-associated cancers (e.g. colon cancer) switch to oxidative metabolism of glucose in the presence of insulin, while others not associated with obesity (e.g. small cell lung cancer) do not [282].

Conversely, there is an increased release of fatty acids [17] into the circulation in obesity, partly due to an increase in adipocyte number and partly due to a decrease in PPARγ, a transcription factor that promotes reesterification [283]. Besides fuelling tumour growth, fatty acids may promote the production of ROS and prostaglandins (which can promote metastasis) by tumour cells via COX2. In murine breast cancer, a shift from glucose metabolism to fatty acid oxidation with a shift to a metastatic phenotype has been noted [284]. Furthermore, adipocytes release fatty acids to nearby cells which in turn increase oxidative capacity and levels of A-FABP [206]. More work is certainly needed to further investigate the role of particular fatty acid families (e.g. unsaturated, short chain) and individual fatty acids (e.g. butyrate) in obesogenic carcinogenesis.

mTOR is a serine/threonine kinase that modulates the balance between cellular metabolism and cell proliferation [285]. mTOR tends to be activated in the obese state, stimulated by leptin and raised insulin, giving increased phosphorylation (activation) of its target, S6K, which promotes protein synthesis [286], priming for growth and proliferation. Upstream of mTOR lies AMP-dependent kinase (AMPK), which inhibits it. AMPK can shift metabolism towards catabolic pathways and away from biosynthesis [287]. This enzyme (and its physiological activator, LKB1, or its pharmacological downstream activator, metformin) can act as tumour suppressors, typically by downregulating mTOR. Indeed, epidemiological studies have shown that metformin may reduce cancer risk and several prospective trials have shown benefits, particularly in breast, colorectal and prostate cancer [288, 289]. Perhaps fortunately, metformin is currently licensed for treatment of type 2 diabetes, which often is associated with obesity. The activity state of AMPK varies in obesity-high leptin levels will inhibit, while adiponectin (which falls in obesity) will activate. Thus, obesity leads to mTOR activation and releases one of the normal brakes on cell growth and proliferation. Furthermore, TP53 stability, as noted above in ‘Evading growth suppressors’*,* is influenced by AMPK and this can have further downstream impacts on metabolic pathways, for instance through TP53-induced glycolysis and apoptosis regulator (TIGAR).

Fat deposition can increase tissue distance from vessels, causing cellular hypoxia. Hypoxia leads to energy production being largely glycolytic (producing lactate) and/or employing reductive glutaminolysis. HIF1α acts as a transcription factor for enzymes that promote a glycolytic phenotype, such as PFK1, GAPDH and aldolase, and isozymes, such as GLUT1, LDH-A and PKM2 [290, 291], while c-myc induces synthesis of glutaminase, the amino acid transporter ASCT2, and LDHA, promoting the use of glutamine as an energy source [292]. Intriguingly, pyruvate kinase M2 (PKM2) also acts as a cotranscriptional activator with HIF1α, leading to a potential positive feedback system [293]. Hypoxia can also influence lipid metabolism, inhibiting desaturation of de novo synthesised lipids by stearoyl-coenzyme A desaturases, e.g. SCD1, in mTOR dysregulated systems [294]. This leads to a dependence on an exogenous supply of unsaturated lipids from the tumour microenvironment.

Serum FGF21 has been shown to be elevated in the serum of overweight and obese humans [54]. Conversely, FGF19 has been reported to be at lower levels in obese patient sera and this too may influence liver metabolism [295]. Adipose-derived exosomes modulate the secretion of FGF21 in the liver, in turn affecting fatty acid oxidation and glucose uptake in multiple tissues [207]. Such exosomes can contain several proteins and RNA species, including miRNAs, many of which can affect metabolic processes. Obesity changes the miRNA content of exosomes in mice; miR-122, miR-192, miR-27a-3p and miR-27b-3p are all increased [72]. These miRNA species can regulate lipid metabolism [296]. When these exosomes were given to lean mice, glucose intolerance and insulin resistance were induced [72]. In breast cancer, exosomes from adipocyte-derived mesenchymal stem cells activate Wnt signalling within tumour cells. Wnt is involved in determining cellular metabolism, by switching between glycolysis and oxidative metabolism, angiogenesis and also regulating cellular metabolism through its effects on expression of transcription factors including c-Myc. c-Myc further influences lipid metabolism via PPARδ [297]. Furthermore, it is worth noting that tumours can also remodel adjacent adipocytes metabolism through exosomal miRNAs, in some cases promoting beige/brown characteristics in breast cancer co-culture (MDA-MB-231, MCF7) [298, 299].

This crosstalk between adipocytes and ASCs has highlighted some other key differences in obesity-related cancer metabolism. Murine models of obesity-accelerated breast cancer highlighted creatine metabolism as a potential key player, with deletion of the rate-limiting enzyme in creatine biosynthesis, glycine amidinotransferase, resulting in reduced tumour growth [300].

Another potentially interesting metabolic modulator in obesity is aquaporin-7 (AQP7). Knockout of AQP7 in mice yields an obese and hyperglycaemic phenotype, secondary to insulin resistance [301]. In breast cancer models, reduced AQP7 protein is associated with altered glutathione metabolism, urea/arginine metabolism and lipid metabolism, as well as reduced local and distant tumour burden [302]. Further work on AQP7 might yield interesting insights.

Circulating metabolites in patients with obesity provide nutrients for cancer cells but the link between obesity and metabolic reprogramming appears to go beyond just providing more fuel. Local and distant crosstalk between adipose tissue and cancer cells appears important, with creatine and glutamine metabolism providing interesting areas for further investigation in obesity-related tumours. We see that metabolic communication can involve miRNAs. Further clarifying how adipose tissue communicates, including through metabolite exchange, may help us understand carcinogenic transformation and subsequent metastasis in obesity.

## Conclusion

We highlight the major and evolving links between obesity and cancer using the hallmarks of cancer as a framework. This work is meant to act as an overview of this complex and evolving area but it is beyond its scope to act as a completely comprehensive reference text. The majority of studies described here were carried out in model organisms and cell lines. More work and innovative study design is required to tease apart obesity and carcinogenesis in the clinic. Indeed, there appears to be paucity in the literature of large prospective cohorts monitoring cytokines and other circulating factors over a range of tissues, and subsequently tumours, in obesity. Whether these obesity-associated factors predispose to specific driver mutations remains to be seen. Perhaps weight-intervention measures or other dietary approaches which are relatively low cost, and could reduce tumorigenesis in obesity as well as other major comorbidities, need greater consideration by public health bodies as the physical and psychological burden of cancer carries significant personal and public health costs. Window of opportunity studies in cancer provide routes to the clinic for novel treatment approaches in patients with obesity. Trials stratifying patients by adipose tissue content, or biomarkers of adiposity that influence carcinogenic processes would be valuable. Particularly repurposing previously approved drugs to target specifically modified pathways in obesity might be fruitful as adjuncts or alternatives to current treatment regimens. Likely candidates come from across specialities and include oestrogen receptor antagonists (e.g. tamoxifen), VEGF signalling inhibitors (e.g. pazopanib), insulin response and mTOR modulators (e.g. metformin), JAK inhibitors (e.g. baricitinib) and even IL-6 receptor antagonists (e.g. tocilizumab). Further work is necessary to resolve the conflicts in the literature surrounding PD-1/PD-L1 inhibitors in obesity, and interventional studies involving the microbiome might be an interesting approach.
